# Ligand Engineering Triggered Efficiency Tunable Emission in Zero-Dimensional Manganese Hybrids for White Light-Emitting Diodes

**DOI:** 10.3390/nano12183142

**Published:** 2022-09-10

**Authors:** Qiqiong Ren, Jian Zhang, Yilin Mao, Maxim S. Molokeev, Guojun Zhou, Xian-Ming Zhang

**Affiliations:** 1 Key Laboratory of Magnetic Molecules and Magnetic Information Materials (Ministry of Education), School of Chemistry and Material Science, Shanxi Normal University, Taiyuan 030031, China; 2 Laboratory of Crystal Physics, Kirensky Institute of Physics, Federal Research Center KSC SB RAS, 660036 Krasnoyarsk, Russia; 3 Research and Development Department, Kemerovo State University, 650000 Kemerovo, Russia; 4 Department of Physics, Far Eastern State Transport University, 680021 Khabarovsk, Russia; 5 Key Laboratory of Interface Science and Engineering in Advanced Material (Ministry of Education), College of Chemistry & Chemical Engineering, Taiyuan University of Technology, Taiyuan 030024, China

**Keywords:** zero-dimensional manganese bromides, steric configurations, tunable emission, white light-emitting diodes

## Abstract

Zero-dimensional (0D) hybrid manganese halides have emerged as promising platforms for the white light-emitting diodes (*w*-LEDs) owing to their excellent optical properties. Necessary for researching on the structure-activity relationship of photoluminescence (PL), the novel manganese bromides (C_13_H_14_N)_2_MnBr_4_ and (C_13_H_26_N)_2_MnBr_4_ are reported by screening two ligands with similar atomic arrangements but various steric configurations. It is found that (C_13_H_14_N)_2_MnBr_4_ with planar configuration tends to promote a stronger electron-phonon coupling, crystal filed effect and concentration-quenching effect than (C_13_H_26_N)_2_MnBr_4_ with chair configuration, resulting in the broadband emission (FWHM = 63 nm) to peak at 539 nm with a large Stokes shift (70 nm) and a relatively low photoluminescence quantum yield (PLQY) (46.23%), which makes for the potential application (LED-1, *R*a = 82.1) in solid-state lighting. In contrast, (C_13_H_26_N)_2_MnBr_4_ exhibits a narrowband emission (FWHM = 44 nm) which peaked at 515 nm with a small Stokes shift (47 nm) and a high PLQY of 64.60%, and the as-fabricated white LED-2 reaches a wide colour gamut of 107.8% National Television Standards Committee (NTSC), thus highlighting the immeasurable application prospects in solid-state display. This work clarifies the significance of the spatial configuration of organic cations in hybrids perovskites and enriches the design ideas for function-oriented low-dimensional emitters.

## 1. Introduction

Low-dimensional organic-inorganic hybrid metal halides (OHMHs) have been widely recognized for their outstanding optical properties, including high-efficiency tunable emissions, near-unity photoluminescence quantum yields (PLQYs), and long decay lifetimes, etc., which are attributed to the strong quantum confinement at the molecular levels and the most convenient radiative recombination of photo-generated excitons [1,2,3,4]. However, the absorption peaks of most OHMHs are located in the ultraviolet (UV) region, which limits the luminous efficiency of white light-emitting diodes (*w*-LEDs) because they cannot be excited by commercial blue chips, thus hindering their industrialization in the fields of solid-state lighting and display. Remarkably, the zero-dimensional (0D) hybrid manganese halides become the preference for blue-light-excited luminescent materials since their photoluminescence excitation (PLE) bands lie in the near-ultraviolet and blue regions, while exhibiting bright photoluminescence (PL) [5,6], and they possess abundant coordination modes and tunable emissions [7,8,9,10,11]. Whether the octahedral coordinated Mn^2+^ ions show intense broadband orange-red emission from the [Mn_3_X_12_]^6−^ inorganic units [12,13,14,15], or the tetrahedral coordinated Mn^2+^ ions exhibit narrowband green emission from the [MnX_4_]^2−^ inorganic units, both of which are attributed to the d-d transition of Mn^2+^ and present application potential in the field of *w*-LEDs [16,17,18,19].

The inorganic units are embedded in organic matrix for OHMHs and are connected to the cations through various molecular interactions, prompting that their structure and PL behaviours can be tuned by engineering the organic cations [7,20]. Different organic cations induce the deformation of inorganic units in varying degrees, resulting in varying crystal field effects, nephelauxetic effects and concentration-quenching effects [21]. Numerous research has exposed that the effect of organic cations on the luminescence properties in 0D hybrid manganese halides. Seshadri et al., and Xia et al., investigated the relationship between structure and PL properties in hybrid manganese halides, and proposed that bulky, rigid, single-protonated cations are in favour of large Mn–Mn distance, thereby leading to high PLQYs [17,22]. Xiao et al., reported and highlighted the effect of organic components with different degrees of conjugation on the optical properties from the view of the band alignment types involving ground state and excited state [7]. Kovalenko et al., found that the PL decay can be accelerated by introducing heavy atoms (e.g., iodine, bromine) in the second coordination sphere of Mn^2+^ [6]. However, these reports ignored the role of spatial configuration of organic components on the fine-tuning PL behaviours and the correspondence between organic components and optical properties of 0D hybrid manganese halides has not been completely established.

Herein, we screen two ligands with similar atomic arrangements but different spatial configurations to synthesize 0D manganese bromides (C_13_H_14_N)_2_MnBr_4_ and (C_13_H_26_N)_2_MnBr_4_ via a simple solution-phase crystallization method. They are crystallized in different space groups and composed of [MnBr_4_]^2−^ twisted tetrahedron and organic matrix with planar and chair configuration, respectively. Experimental and theoretical researches indicate that the organic cation with planar configuration tend to induce stronger electron-phonon coupling, crystal filed effect and concentration-quenching effect than chair configuration in these 0D manganese bromides, thereby finely tuning PL behaviours, including emission bandwidth, peak position, Stokes shift, lifetimes and PLQYs, as well as the luminescence mechanism was discussed by combining the Tunabe-Sugano (T-S) energy diagram. The *w*-LEDs devices were fabricated using the above manganese bromides as green components; it is concluded that LED-1 based on (C_13_H_14_N)_2_MnBr_4_ with a high *R*_a_ of 82.1 is suitable for solid-state lighting, while LED-2 based on (C_13_H_26_N)_2_MnBr_4_ with a wide colour gamut of 107.8% NTSC holds unprecedented promising for use in solid-state displays. Our work demonstrates that the organic cations with different spatial configurations are able to trigger tunable emission in 0D Manganese hybrids, and the design principle provides a new idea for future research on white light-emitting materials.

## 2. Experimental

### 2.1. Materials and Preparation

*N*-Methyldiphenylamine (C_13_H_13_N, 98%, Aladdin, Shanghai, China), *N,N*-Dicyclohexylmethylamine (C_13_H_25_N, 98%, Aladdin, Shanghai, China), manganese bromide tetrahydrate (MnBr_2_·4H_2_O, 98%, Aladdin, Shanghai, China), hydrobromic acid (HBr, 40%, Aladdin, Shanghai, China) and ethanol (C_2_H_5_OH, 99.7%, Guangfu, Tianjin, China). MnBr_2_·4H_2_O needs to be heated at 120 °C for 6 h to remove the crystal water for later use.

The single-crystals of (C_13_H_14_N)_2_MnBr_4_ and (C_13_H_26_N)_2_MnBr_4_ were synthesized by a simple solution-phase crystallization method. First, C_13_H_13_N (0.3665 g, 2 mmol) or C_13_H_25_N (0.3907 g, 2 mmol) was dissolved in 1.5 mL HBr for protonation. Then MnBr_2_ (0.2148 g, 1 mmol) and 5 mL C_2_H_5_OH were added to the above protonated solution, and heated and stirred at 75 °C until forming a clear solution. After the solution was naturally cooled to room temperature, yellow (C_13_H_14_N)_2_MnBr_4_ and green (C_13_H_26_N)_2_MnBr_4_ bulky crystals were precipitated overnight. Finally, the crystals were washed several times with acetone and dried in a vacuum oven.

### 2.2. Characterization

Single-crystal X-ray diffraction (SCXRD) data of (C_13_H_14_N)_2_MnBr_4_ and (C_13_H_26_N)_2_MnBr_4_ were collected by using an Agilent Technologies Gemini EOS (Palo Alto, CA, USA) diffractometer at 298 K using Mo Kα radiation (*λ* = 0.71073 Å). Powder X-ray diffraction (PXRD) data of (C_13_H_14_N)_2_MnBr_4_ and (C_13_H_26_N)_2_MnBr_4_ for Rietveld analysis was collected at room temperature with a Bruker D8 ADVANCE powder diffractometer EOS (Karlsruhe, Germany) (Cu-Kα radiation) and linear VANTEC detector. The step size of 2θ was 0.011°, and the counting time was 2 s per step. Rietveld structure refinements were performed by using TOPAS 4.2. PL, PLE spectra, PL decay curves and PLQYs were measured by an Edinburgh FLS920 fluorescence spectrometer EOS (Edinburgh, UK) with a picosecond pulsed diode laser. Temperature-dependent emission spectra were measured on the same spectrophotometer installed with a heating apparatus as the heating source. Morphology observation and elemental mappings were conducted by a scanning electron microscope (SEM, JEOL JSM-6510, EOS, Peabody, MA, USA). UV-vis absorption curves were recorded on a TU-1901 Ultraviolet spectrometer EOS (Beijing, China) at room temperature, in which BaSO_4_ was used as the standard reference. CIE chromaticity coordinates were calculated using the CIE calculator software based on the emission spectra excited at 450 nm. The emission spectra, correlated colour temperature (CCT), luminous efficacy, and CIE coordinates of *w*-LEDs were performed on the integrating sphere spectroradiometer system (ATA-100, Everfine, EOS, Hangzhou, Cina).

### 2.3. Computational Methods

The electronic band structure and density of state (DOS) were calculated by CASTEP based on plane-wave pseudopotential density functional theory (DFT) [23]. Perdew-Burke-Ernzerhof (PBE) functionals in the form of general gradient approximation (GGA) were used for electronic structure calculations [24]. A kinetic energy cut off value of 450 eV and a Monkhorst-pack k-point mesh spanning less than 0.03 Å^−1^ in the Brillouin zone were chosen.

## 3. Results and Discussion

**Figure 1:**
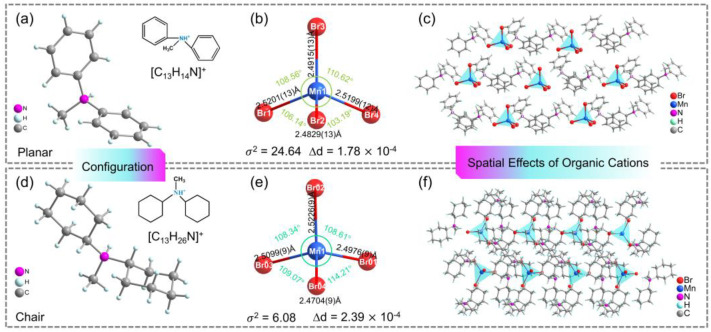
(**a**,**d**) The chemical structures of organic cations [C_13_H_14_N]^+^ and [C_13_H_26_N]^+^. (**b**,**e**) Ball and stick models of inorganic units and structural information including bond length, bond angles and twist degrees. (**c**,**f**) Crystal structures of (C_13_H_14_N)_2_MnBr_4_ and (C_13_H_26_N)_2_MnBr_4_.

As shown in Figure 1a,d, two ligands with similar atomic arrangements but different spatial configurations were screened out to synthesize hybrid manganese bromides (C_13_H_14_N)_2_MnBr_4_ and (C_13_H_26_N)_2_MnBr_4_ with MnBr_2_ via a simple solution-phase crystallization method. The crystal structures resolved by SCXRD technique show that both (C_13_H_14_N)_2_MnBr_4_ and (C_13_H_26_N)_2_MnBr_4_ correspond to typical 0D “host-guest” structures. That is, central metal Mn^2+^ ions are coordinated with four Br^−^ anions to form twisted tetrahedral [MnBr_4_]^2−^ inorganic units and all inorganic components are periodically dispersive embedded in insulating organic cations (Figure 1c,f). Different organic cations induce the distinct packing and deformation of anions. Therefore, (C_13_H_14_N)_2_MnBr_4_ and (C_13_H_26_N)_2_MnBr_4_ are crystallized in triclinic space group *P*1 and monoclinic space group *P*2_1_/*c*, respectively. As shown in Figure 1b,e, two different [MnBr_4_]^2−^ inorganic units exhibit different distortion degree. The distances of four Mn–Br bonds are in the range of 2.4829(13)–2.5201(13) Å and the Br–Mn–Br bond angles ranged from 103.19(5) to 117.83(6)° for (C_13_H_14_N)_2_MnBr_4_. However, this structural information is in the range of 2.4704(9)–2.5226(9) Å and 107.07(3) to 114.21(4)° for (C_13_H_26_N)_2_MnBr_4_. The crystallographic information files (CIFs) are shown in the Supporting Information (SI), and the main bond lengths and bond angles are shown in Table S1 and Table S2, respectively. The bond length distortion (∆*d*) and bond angle variance (*σ*^2^) of individual [MnBr_4_]^2−^ are calculated by the following formulas [25,26]:

$$\Delta d=1/4\overset{4}{\underset{i=1}{\sum }}{\left(\left(d_{i}-d_{0}\right)/d_{0}\right)}^{2}$$

where $d_{0}$ represents the average Mn–Br bond length and $d_{i}$ are four individual lengths of Mn–Br bond.

$$\sigma^{2}=1/5\overset{6}{\underset{i=1}{\sum }}{\left(\theta_{i}-109.47\right)}^{2}$$

where $\theta_{i}$ refer to the individual Br–Mn–Br angles. The Δd values of (C_13_H_14_N)_2_MnBr_4_ and (C_13_H_26_N)_2_MnBr_4_ are 1.78 × 10^−4^ and 2.39 × 10^−4^, respectively. The $\sigma^{2}$ values are 24.64 and 6.08. It is worth noting that the difference in bond length distortion is small and can be ignored, since the large distinction in the bond angle variance maybe the underlying reason for the disparate optical properties of (C_13_H_14_N)_2_MnBr_4_ and (C_13_H_26_N)_2_MnBr_4_.

**Figure 2:**
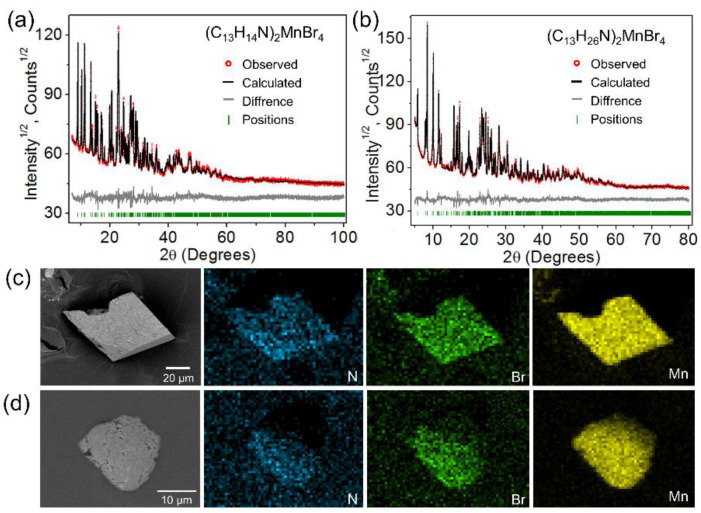
Rietveld refinements of PXRD patterns for (**a**) (C_13_H_14_N)_2_MnBr_4_, (**b**) (C_13_H_26_N)_2_MnBr_4_. SEM images and EDS elemental mapping images showing the homogeneous distribution of N, Mn and Br in compounds for (**c**) (C_13_H_14_N)_2_MnBr_4_ and (**d**) (C_13_H_26_N)_2_MnBr_4_.

The phase purity and crystallinity of (C_13_H_14_N)_2_MnBr_4_ and (C_13_H_26_N)_2_MnBr_4_ powders were monitored by PXRD, and the results are shown in Figure S1. All peaks were indexed by triclinic cell (*P*1) and monoclinic cell (*P*2_1_/*c*) with parameters close to those obtained from a single crystal experiment, respectively. Therefore, these structures were considered as a starting model for Rietveld refinement, as shown in Figure 2a,b, which were performed using TOPAS 4.2. The refinement results were stable and gave low R-factors. The main parameters of processing and refinement of (C_13_H_14_N)_2_MnBr_4_ and (C_13_H_26_N)_2_MnBr_4_ were listed in Table S3. The elemental mapping images (Figure 2c,d) indicate that the elements N, Br and Mn are evenly distributed on the above manganese bromides.

**Figure 3:**
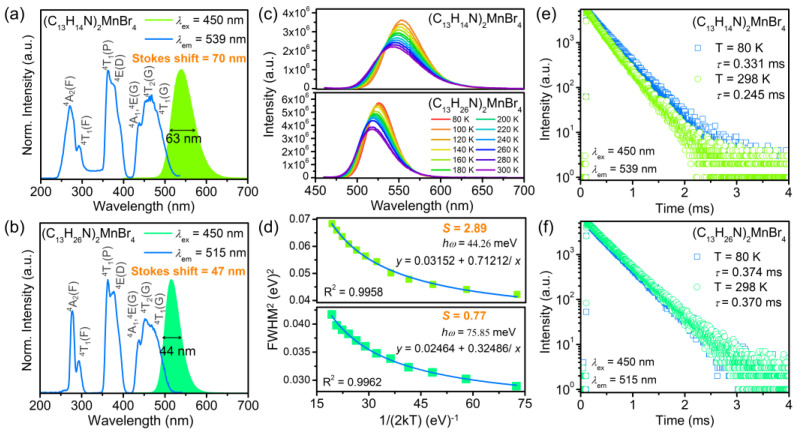
Normalized PLE and PL spectra of (**a**) (C_13_H_14_N)_2_MnBr_4_ and (**b**) (C_13_H_26_N)_2_MnBr_4_. (**c**) Temperature-dependent PL spectra of (C_13_H_14_N)_2_MnBr_4_ and (C_13_H_26_N)_2_MnBr_4_ under 450 nm excitation in the range from 80 to 300 K. (**d**) FWHM^2^ fitting curves of (C_13_H_14_N)_2_MnBr_4_ and (C_13_H_26_N)_2_MnBr_4_ as a function of 1/(2kT). Time-resolved PL decay curves of (**e**) (C_13_H_14_N)_2_MnBr_4_ and (**f**) (C_13_H_26_N)_2_MnBr_4_ at 298 K and 80 K.

As shown in Figure S2b,c, the manganese bromides of (C_13_H_14_N)_2_MnBr_4_ and (C_13_H_26_N)_2_MnBr_4_ with yellow-green body colour exhibit bright yellow-green fluorescence under a 365 nm UV lamp, and the corresponding CIE coordinates are depicted in Figure S2a. To further reveal their PL properties, the PLE and PL spectra of (C_13_H_14_N)_2_MnBr_4_ and (C_13_H_26_N)_2_MnBr_4_ are initially investigated in Figure 3a,b. Both manganese bromides exhibit similar excitation bands attributed to the d–d transition of tetrahedrally coordinated Mn^2+^ centres and can be excited by blue light, but they possess distinct emission spectra. (C_13_H_14_N)_2_MnBr_4_ displays a broadband emission peaked at 539 nm with a full width at half maximum (FWHM) of 63 nm and the Stokes shift of 70 nm, which is wider than most 0D hybrid manganese halides [19,27,28]. In contrast, the emission peak of (C_13_H_26_N)_2_MnBr_4_ appears at 515 nm, with a FWHM of 44 nm and the Stokes shift of 47 nm. Meanwhile, we synthesized the powder chlorides of (C_13_H_14_N)_2_MnCl_4_ and (C_13_H_26_N)_2_MnCl_4_ using the above two ligands and they exhibit the same effect and difference on their PL properties as (C_13_H_14_N)_2_MnBr_4_ and (C_13_H_26_N)_2_MnBr_4_ (Figure S3). We consider that the essence for the PL difference may be caused by the different spatial configuration of organic components in above manganese bromides. The ligand C_13_H_13_N contains two planar benzene rings with little steric hindrance. However, the twelve carbon atoms are not in the same plane for C_13_H_25_N, so that there is a larger steric hindrance. Proceeding from (C_13_H_14_N)_2_MnBr_4_ to (C_13_H_26_N)_2_MnBr_4_, the increasing cation volume and the steric hindrance not only shrink the free space for the atom movement or the cation/anion rotation, but also inhibit the lattice distortion [29,30]. Therefore, (C_13_H_14_N)_2_MnBr_4_ produce stronger electron-phonon coupling and crystal field strength than (C_13_H_26_N)_2_MnBr_4_, resulting in a broadband emission with large Stokes shift.

Figure 3c depicts the temperature-dependent PL spectra of (C_13_H_14_N)_2_MnBr_4_ and (C_13_H_26_N)_2_MnBr_4_ in the range of 80–300 K. The PL intensity decreases with the increasing temperature, which is consistent with the PL quenching behaviour caused by thermally activated non-radiative recombination. Meanwhile, the emission peak positions have a blue-shift. It is speculated that the reason for this phenomenon is that the thermally induced lattice expansion weakens the crystal field strength, or reduces the energy loss due to the spin-spin coupling between localized neighbouring Mn^2+^ ions [16]. Furthermore, the FWHM versus temperature (Figure 3d) can reveal the origin of the PL differences in above manganese bromides. The Huang-Rhys factor *S* defines the degree of electron–phonon coupling; it can be solved by the following equation [18,31,32]:

$$\mathrm{FWHM}=2.36\hbar \omega \sqrt{S\;coth\left(\frac{\hbar \omega }{2kT}\right)\;\;}$$

where *ω* is the phonon frequency, ħω is the maximum phonon energy, *k* is the Boltzmann constant, *S* is Huang-Rhys factor, and $coth\left(x\right)=\frac{e^{x}+e^{-x}}{e^{x}-e^{-x}}=1+\frac{2}{e^{2x}-1}$. When $\frac{\hbar \omega }{kT}$ is small enough, $e^{\frac{\hbar \omega }{kT}}-1\approx \frac{\hbar \omega }{kT}$, it can be obtained the following equation:

$${\mathrm{FWHM}}^{2}=5.57{\left(\hbar \omega \right)}^{2}S\left(1+\frac{2}{e^{\frac{\hbar \omega }{kT}}-1}\right)=5.57{\left(\hbar \omega \right)}^{2}S\left(1+\frac{1}{\frac{\hbar \omega }{2kT}}\right)$$

Further written as:

$${\mathrm{FWHM}}^{2}=a+\frac{b}{\frac{1}{2kT}}$$

where *a* = 5.57 × *S* × (ħω)^2^ and *b* = 5.57 × *S* × (ħω).

The obtained *S* factor is 2.89 and phonon energy ħω phonon is 44.26 meV for (C_13_H_14_N)_2_MnBr_4_, while for (C_13_H_26_N)_2_MnBr_4_, it corresponds to *S* = 0.77, ħω = 75.85 meV. The higher *S* factor indicates that there is a stronger electron-phonon coupling in (C_13_H_14_N)_2_MnBr_4_, which is favourable for the formation of broadband emission with large Stokes shift.

In addition, the crystal field strength plays a key role in affecting the PL properties of manganese bromides. Previous references reported that the crystal field strength is related to the polyhedral distortion, and the increasing distortion leads to strong crystal field splitting and low position of the lowest 3d excited energy levels, resulting in the red-shift of the emission band [33,34,35,36]. The $\sigma^{2}$ values of (C_13_H_14_N)_2_MnBr_4_ and (C_13_H_26_N)_2_MnBr_4_ are 24.64 and 6.08, respectively, as calculated above, indicating that the larger bond angle distortion contributes to a red-shift of the emission peak from 515 nm to 539 nm.

To verify the electronic transition process, we measured the lifetimes excited at 450 nm and monitored at 539 nm and 515 nm, respectively. As shown in Figure 3e,f, the PL decay curves can be fitted with a single order exponential equation:

$$I\left(t\right)=I_{0}+A\;\exp\;\left(-t/\tau \right)$$

where *I*(*t*) and *I*_0_ are the luminescence intensity at time *t* and *t* ≫ *τ*, respectively. *A* is a constant, and *τ* is the decay time for an exponential component. At 298 K, the lifetimes are on millisecond scale, and the values are determined to be 0.245 ms and 0.370 ms, respectively, which are close to the previously reported lifetimes of hybrid manganese bromides, demonstrating that these emissions belong to the d–d transition (^4^T_1_–^6^A_1_) of Mn^2+^ [17,37,38]. At 80 K, the lifetimes are prolonged, but the variation tendency of (C_13_H_14_N)_2_MnBr_4_ is greater than that of (C_13_H_26_N)_2_MnBr_4_, which may be because that (C_13_H_14_N)_2_MnBr_4_ has more vibration options. In addition, PLQY is also affected by the spatial configuration of organic cations in these manganese bromides. Figure S4 shows that the [C_13_H_14_N]^+^ cations containing benzene ring planar configuration generate a short average distance between the nearest [MnBr_4_]^2−^ is 8.5934 Å, while the chair configuration of [C_13_H_26_N]^+^ cations with greater steric hindrance produce a longer Mn–Mn average distance is 9.6703 Å. Accordingly, the concentration-quenching effect of (C_13_H_26_N)_2_MnBr_4_ is weakened, resulting in PLQYs of 46.23% and 64.60% for (C_13_H_14_N)_2_MnBr_4_ and (C_13_H_26_N)_2_MnBr_4_, respectively.

**Figure 4:**
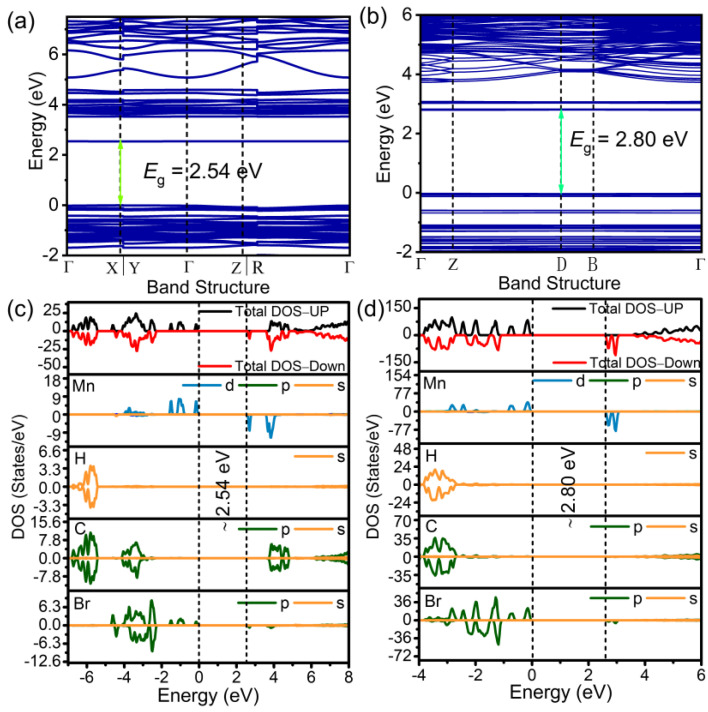
Electronic energy band structures of (**a**) (C_13_H_14_N)_2_MnBr_4_ and (**b**) (C_13_H_26_N)_2_MnBr_4_. The total and orbital projection of partial density of states of (**c**) (C_13_H_14_N)_2_MnBr_4_ and (**d**) (C_13_H_26_N)_2_MnBr_4_.

To further reveal the photo-physical properties of (C_13_H_14_N)_2_MnBr_4_ and (C_13_H_26_N)_2_MnBr_4_ composed of organic moieties with different spatial configurations. The theoretical calculations involving electronic band structure and densities of states (DOS) are conducted based on the density functional theory. As shown in Figure 4a,b, the calculated band gap of (C_13_H_14_N)_2_MnBr_4_ and (C_13_H_26_N)_2_MnBr_4_ are 2.54 eV and 2.80 eV, respectively, which are coincide with the observed optical absorption spectra (Figure S5). It is worth noting that the (C_13_H_14_N)_2_MnBr_4_ has a lower conduction band level compared to (C_13_H_26_N)_2_MnBr_4_, resulting from the higher degree of conjugation of organic component, which is also responsible for the red-shift of the emission peak of (C_13_H_14_N)_2_MnBr_4_. Both the frontier orbitals are flat and dispersion-less, indicating that the photoelectrons are localized for above manganese bromides. This is because that the isolated [MnBr_4_]^2−^ tetrahedrons separated by bulky organic cations have weak interactions, resulting in spatial confinement and electronic confinement effects. Figure 4c,d show the DOS projected onto the constituent elements in (C_13_H_14_N)_2_MnBr_4_ and (C_13_H_26_N)_2_MnBr_4_. The valence band maximum (VBM) states and the conduction band minimum (CBM) states are mainly composed of Mn 3d and Br 3p orbitals, indicating that the electronic transition process related to luminescence occurs within the [MnBr_4_]^2−^ inorganic units, and the VBM- and CBM-associated charge densities (Figure S6) also confirm this mechanism.

**Figure 5:**
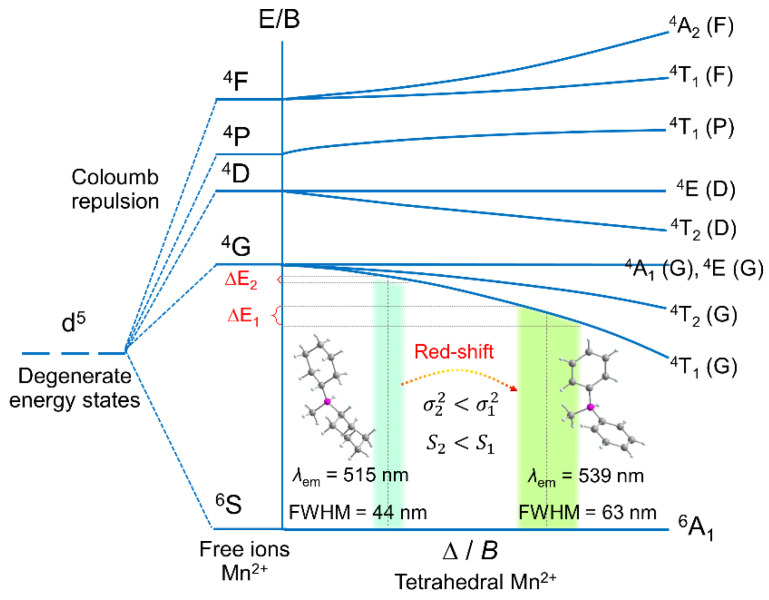
Tanabe–Sugano energy-level diagram of Mn^2+^ ions in tetrahedral crystal field.

According to the electron transition process, we employ the T-S energy diagram to visualize the effect of spatial configuration of organic ligands on PL behaviours. It is well known that the Mn^2+^ ions possess the outermost electron configuration of 3d^5^, and the original five degenerate d orbitals of free Mn^2+^ are split into multiple energy levels under the electrostatic field effect generated by ligands. In the tetrahedral field, the ground state is denoted as ^6^A_1_, the excited states of ^4^F, ^4^P, ^4^D and ^4^G correspond to the excited energy levels in the UV (200–400 nm) and blue (400–500 nm) regions, respectively [6,7]. The electrons located at the ground state are transferred to different excited states under excitation, and then transferred to the lowest excited state energy level ^4^T_1_ through non-radiative relaxation, and finally the electrons return to the ground state with the release of photons. In the T-S energy diagram, the strength of the crystal field is represented by the abscissa (∆) and the energy difference between excited states ^4^T_2_ and ^4^T_1_. The stronger the crystal field strength, the more obvious the splitting of ^4^T_2_ and ^4^T_1_, making the larger values of E [^4^T_2_]–E[^4^T_1_]. In view of this, the luminescent mechanism is depicted in Figure 5. (C_13_H_14_N)_2_MnBr_4_ and (C_13_H_26_N)_2_MnBr_4_ are arranged in the region of strong crystal field and weak crystal field, respectively. (C_13_H_14_N)_2_MnBr_4_ occupies a wider range on the abscissa than (C_13_H_26_N)_2_MnBr_4_, due to the larger electron-phonon coupling coefficient for (C_13_H_14_N)_2_MnBr_4_, resulting in the crystal field strength varies greatly with lattice vibrations. Reflected on the vertical coordinates, it is shown that (C_13_H_14_N)_2_MnBr_4_ has a larger ∆E than (C_13_H_26_N)_2_MnBr_4_, that is, (C_13_H_14_N)_2_MnBr_4_ exhibits a broadband emission with a large Stokes shift.

**Figure 6:**
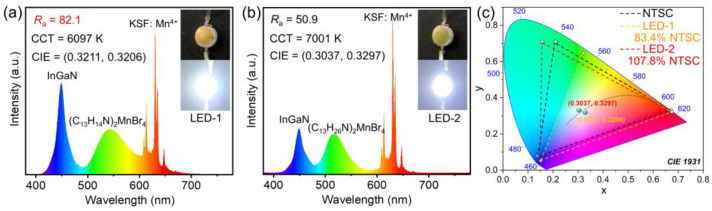
Emission spectrum of *w*-LEDs based on (**a**) (C_13_H_14_N)_2_MnBr_4_ and (**b**) (C_13_H_26_N)_2_MnBr_4_. The insets show the photographs of *w*-LEDs in the daylight (top) and working at 20 mA (bottom). (**c**) CIE 1931 colour coordinates of fabricated LED-1 (orange dotted line), LED-2 (red dotted line) and the NTSC standard (black dotted line).

To further evaluate their practical applications, the *w*-LEDs (LED-1 and LED-2) were encapsulated by coating (C_13_H_14_N)_2_MnBr_4_/(C_13_H_26_N)_2_MnBr_4_ and KSF:Mn^4+^ red phosphors on a commercial InGaN blue chip (450 nm), respectively. As shown in Figure 6, LED-1 presents the standard white-light emission with a relatively high colour-rendering index (CIR) of *R*a = 82.1, and a CIE chromaticity coordinate of (0.3211, 0.3206). LED-2 presents a cool white-light emission with a wide colour gamut of 107.8% NTSC in the CIE 1931 colour space, and a CIE chromaticity coordinate of (0.3037, 0.3297). It is concluded that (C_13_H_14_N)_2_MnBr_4_ is more suitable for solid-state lighting, while (C_13_H_26_N)_2_MnBr_4_ is unprecedentedly promising as a narrow-band green emitter for solid-state displays. The emission spectra of LED-1 and LED-2 under different drive current different current (20−120 mA) in Figure S7 suggests that the *w*-LEDs possess excellent colour stability. Of course, we also evaluated the thermal quenching behaviour of (C_13_H_14_N)_2_MnBr_4_ and (C_13_H_26_N)_2_MnBr_4_. As shown in Figure S8, the PL intensity decreases with increasing temperature. The PL intensity of (C_13_H_14_N)_2_MnBr_4_ and (C_13_H_26_N)_2_MnBr_4_ at 373 K remain around 79.86% and 84.84% of that at room temperature, respectively, suggesting that the above manganese bromides have relatively good thermal stability.

## 4. Conclusions

In summary, the two new 0D manganese bromides were developed by using organic cations with different spatial configuration. (C_13_H_14_N)_2_MnBr_4_ exhibits a broadband emission peaked at 539 nm with a FWHM of 63 nm and a Stokes shift of 70 nm, while (C_13_H_26_N)_2_MnBr_4_ shows a narrowband emission peaked at 515 nm with a FWHM of 44 nm and a Stokes shift of 47 nm, which is ascribed to that the ligand of C_13_H_13_N with planar configuration induces a stronger electron-phonon coupling (*S* = 2.89) and crystal field strength ($\sigma^{2}$ = 24.64) than the ligand of C_13_H_25_N with chair configuration. DFT calculations reveal that (C_13_H_14_N)_2_MnBr_4_ possesses a narrower bandgap compared to (C_13_H_26_N)_2_MnBr_4_, resulting in the red-shift of the emission peak of (C_13_H_14_N)_2_MnBr_4_. Moreover, the steric effect of organic cations on PL behaviours in manganese halides is comprehensively reflected in T-S energy diagram. The as-fabricated white LED-1 based on (C_13_H_14_N)_2_MnBr_4_ with a high CIR of *R*a = 82.1 is suitable for solid-state lighting, while the as-fabricated white LED-2 based on (C_13_H_26_N)_2_MnBr_4_ with a wide colour gamut of 107.8% NTSC in the CIE 1931 colour space presents an unprecedentedly promising utility for solid-state display.

## Data Availability

The data presented in this study are available on request from the corresponding author.

## Supplementary Materials

The following supporting information can be downloaded at: https://www.mdpi.com/article/10.3390/nano12183142/s1, Figure S1: PXRD patterns and standard diffraction pattern of (C_13_H_14_N)_2_MnBr_4_ and (C_13_H_26_N)_2_MnBr_4_; Figure S2: CIE chromaticity diagrams of (C_13_H_14_N)_2_MnBr_4_ and (C_13_H_26_N)_2_MnBr_4_ and the photographs with UV off and on; Figure S3: Normalized PLE and PL spectra and time-resolved PL decay curves of (C_13_H_14_N)_2_MnCl_4_ and (C_13_H_26_N)_2_MnCl_4_ at 298 K; Figure S4: The schematic diagrams of Mn···Mn distance; Figure S5: Absorption spectra of (C_13_H_14_N)_2_MnBr_4_ and (C_13_H_26_N)_2_MnBr_4_; Figure S6: VBM- and CBM-associated charge densities of (C_13_H_14_N)_2_MnBr_4_ and (C_13_H_26_N)_2_MnBr_4_; Figure S7: PL spectra of the w-LED devices under different current (20−120 mA); Figure S8: Temperature-dependent PL spectra of (C_13_H_14_N)_2_MnBr_4_ and (C_13_H_26_N)_2_MnBr_4_ ranging from 298 K to 423 K; Tables S1 and S2: The bond length (Å) and the bond angles (°) of (C_13_H_14_N)_2_MnBr_4_ and (C_13_H_26_N)_2_MnBr_4_; Table S3: Main parameters of processing and refinement of (C_13_H_14_N)_2_MnBr_4_ and (C_13_H_26_N)_2_MnBr_4_.
