# Supplementary material for: Ligand Engineering Triggered Efficiency Tunable Emission in Zero-Dimensional Manganese Hybrids for White Light-Emitting Diodes
Source: Nanomaterials (Basel). 2022 Sep 10;12(18):3142. doi: 10.3390/nano12183142 (PMC9501502; doi:10.3390/nano12183142)
Supplement: Supplementary file 1 [file nanomaterials-12-03142-s001.zip › nanomaterials-1890324-supplementary.pdf]

nanomaterials

Supplementary Materials

Ligand Engineering Triggered Efficiency Tunable Emission in Zero-Dimensional Manganese Hybrids for White Light-Emitting Diodes

Qiqiong Ren [1], Jian Zhang [1], Yilin Mao [1], Maxim S. Molokeev [2,3,4], Guojun Zhou [1,*] and Xian-Ming Zhang [1,5,*]

[1] Key Laboratory of Magnetic Molecules and Magnetic Information Materials (Ministry of Education), School of Chemistry and Material Science, Shanxi Normal University, Taiyuan 030031, China
[2] Laboratory of Crystal Physics, Kirensky Institute of Physics, Federal Research Center KSC SB RAS, 660036 Krasnoyarsk, Russia
[3] Research and Development Department, Kemerovo State University, 650000 Kemerovo, Russia
[4] Department of Physics, Far Eastern State Transport University, 680021 Khabarovsk, Russia
[5] Key Laboratory of Interface Science and Engineering in Advanced Material (Ministry of Education), College of Chemistry & Chemical Engineering, Taiyuan University of Technology, Taiyuan 030024, China
* Correspondence: zhougj@sxnu.edu.cn (G.Z.); zhangxm@dns.sxnu.edu.cn (X.-M.Z.)

Citation: Ren, Q.; Zhang, J.; Mao, Y.; Molokeev, M.S.; Zhou, G.; Zhang, X.-M. Ligand Engineering Triggered Efficiency Tunable Emission in Zero-Dimensional Manganese Hybrids for White Light-Emitting Diodes. *Nanomaterials* **2022**, *12*, 3142. https://doi.org/10.3390/nano12183142

Academic Editors: Aurora Rizzo and Bouchta Sahraoui

Received: 14 August 2022
Accepted: 7 September 2022
Published: 10 September 2022

Publisher's Note: MDPI stays neutral with regard to jurisdictional claims in published maps and institutional affiliations.

Copyright: © 2022 by the authors. Licensee MDPI, Basel, Switzerland. This article is an open access article distributed under the terms and conditions of the Creative Commons Attribution (CC BY) license (https://creativecommons.org/licenses/by/4.0/).

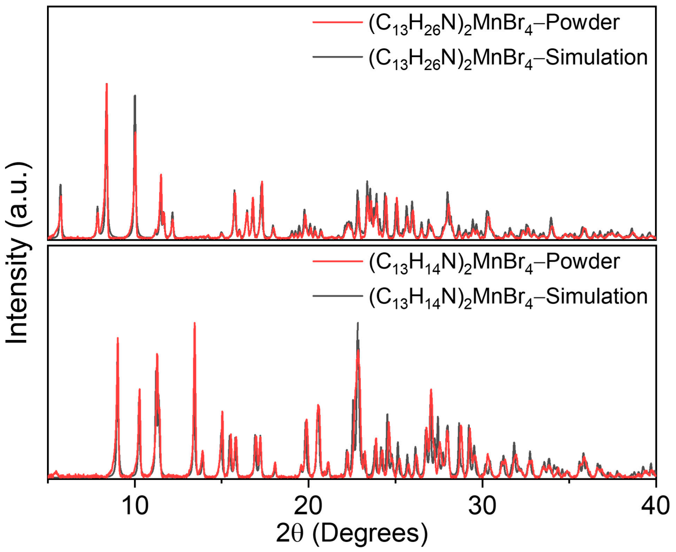

Figure S1. PXRD patterns and standard diffraction pattern of $(C_{13}H_{14}N)_2MnBr_4$ and $(C_{13}H_{26}N)_2MnBr_4$.

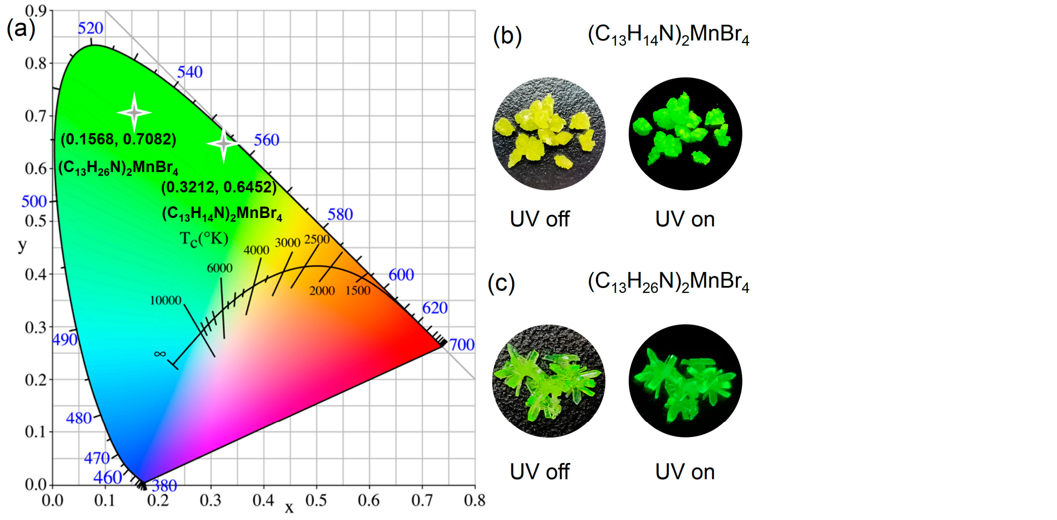

Figure S2. (**a**) The CIE chromaticity diagrams of $(C_{13}H_{14}N)_2MnBr_4$ and $(C_{13}H_{26}N)_2MnBr_4$. (**b, c**) The photographs of $(C_{13}H_{14}N)_2MnBr_4$ (top) and $(C_{13}H_{26}N)_2MnBr_4$ (bottom) with UV off and on.

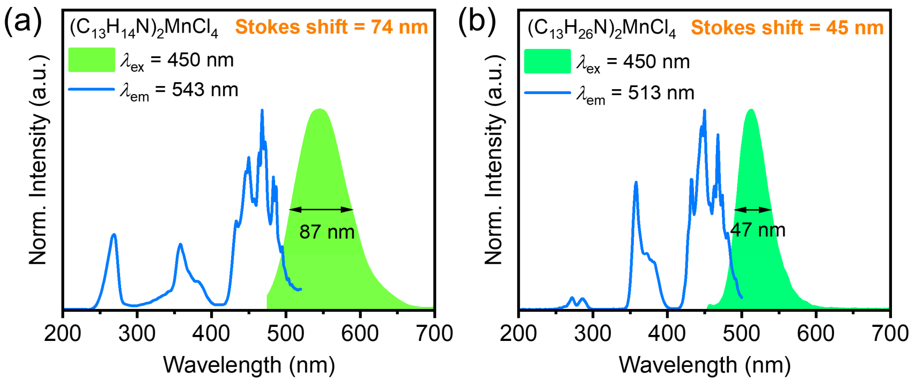

Figure S3. Normalized PLE and PL spectra of (**a**) $(C_{13}H_{14}N)_2MnCl_4$ and (**b**) $(C_{13}H_{26}N)_2MnCl_4$.

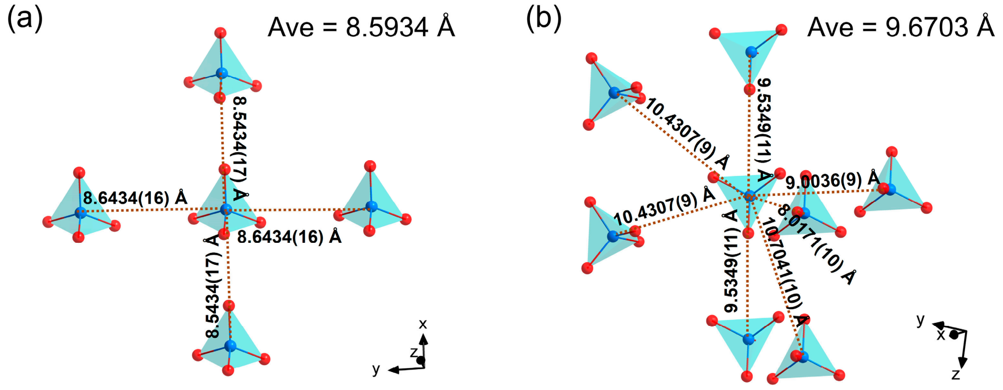

Figure S4. The schematic diagrams of Mn···Mn distance in (**a**) $(C_{13}H_{14}N)_2MnBr_4$ and (**b**) $(C_{13}H_{26}N)_2MnBr_4$.

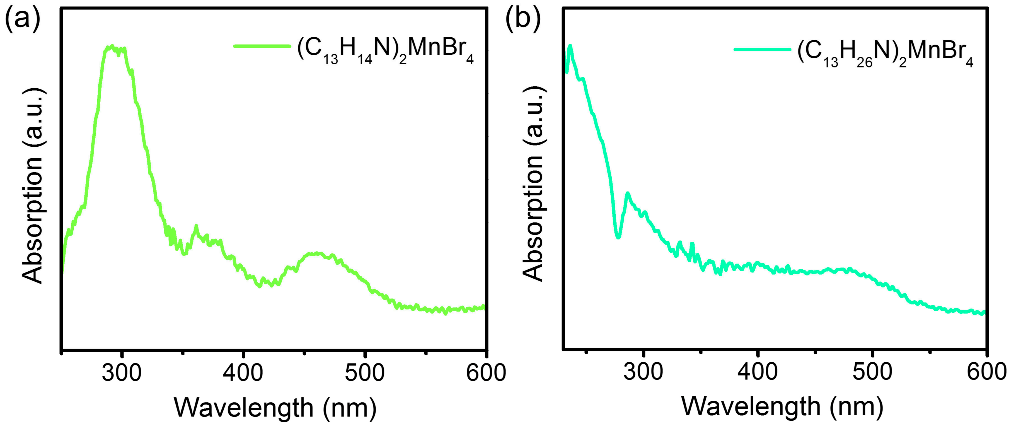

Figure S5. Absorption spectra of (**a**) $(C_{13}H_{14}N)_2MnBr_4$ and (**b**) $(C_{13}H_{26}N)_2MnBr_4$.

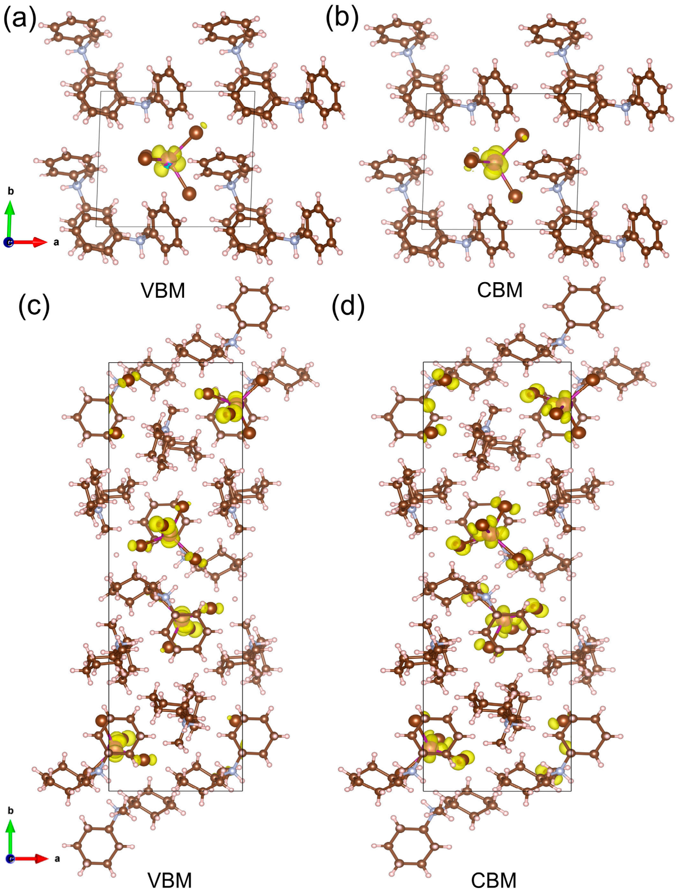

Figure S6. VBM- and CBM-associated charge densities of (**a**, **b**) $(C_{13}H_{14}N)_2MnBr_4$ and (**c**, **d**) $(C_{13}H_{26}N)_2MnBr_4$.

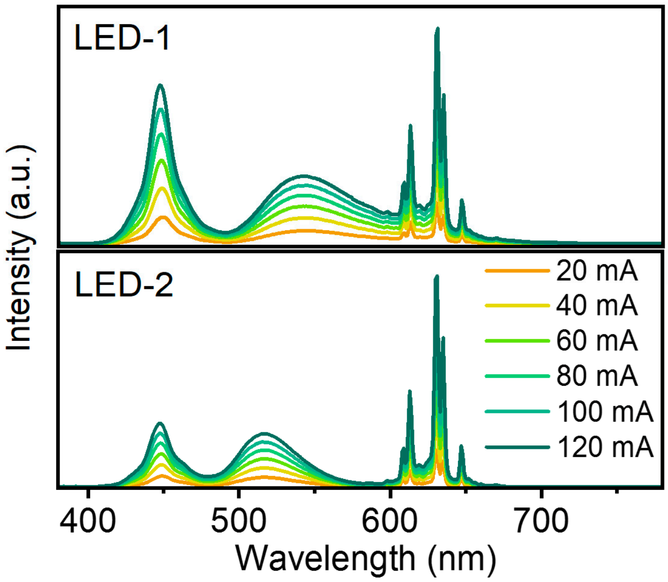

Figure S7. PL spectra of the WLED devices excited at different current (20–120 mA).

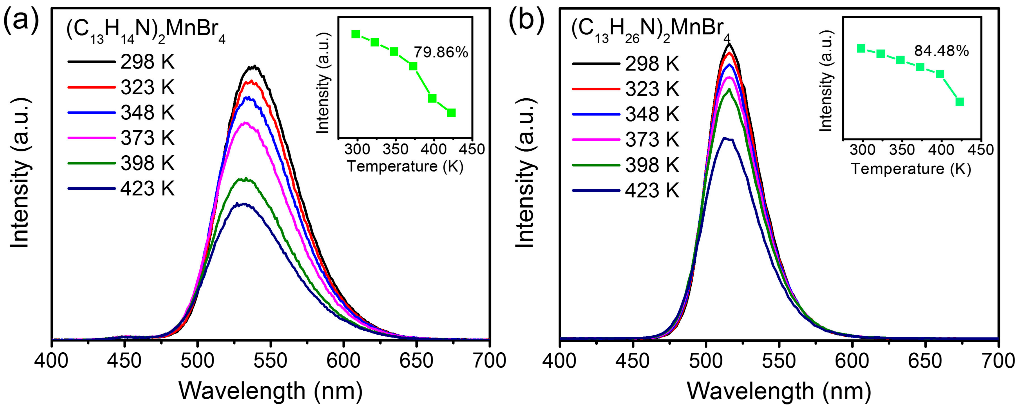

Figure S8. Temperature-dependent PL spectra of (**a**) $(C_{13}H_{14}N)_2MnBr_4$ and (**b**) $(C_{13}H_{26}N)_2MnBr_4$ under 450 nm excitation ranging from 298 K to 423K.

Table S1. The bond length (Å) of $(C_{13}H_{14}N)_2MnBr_4$ and $(C_{13}H_{26}N)_2MnBr_4$.

**$(C_{13}H_{14}N)_2MnBr_4$**			
Atom–Atom	Length/Å	Atom–Atom	Length/Å
Br (3)–Mn (1)	2.4915 (13)	C (16)–C (15)	1.384 (14)
Br (1)–Mn (1)	2.5201 (13)	C (9)–C (8)	1.369 (14)
Br (4)–Mn (1)	2.5199 (12)	C (9)–C (10)	1.361 (13)
Br (2)–Mn (1)	2.4829 (13)	C (14)–C (15)	1.376 (12)
N (1)–C (19)	1.486 (9)	C (6)–C (5)	1.392 (19)
N (1)–C (20)	1.515 (11)	C (23)–C (22)	1.398 (13)
N (1)–C (21)	1.496 (9)	C (23)–C (24)	1.369 (19)
N (2)–C (1)	1.501 (9)	C (11)–C (10)	1.389 (16)
N (2)–C (7)	1.491 (12)	C (11)–C (12)	1.37 (2)
N (2)–C (8)	1.479 (9)	C (5)–C (4)	1.37 (2)
C (18)–C (19)	1.371 (10)	C (8)–C (13)	1.383 (14)
C (18)–C (17)	1.397 (12)	C (22)–C (21)	1.375 (10)
C (1)–C (2)	1.332 (13)	C (21)–C (26)	1.361 (13)
C (1)–C (6)	1.360 (13)	C (26)–C (25)	1.403 (15)
C (19)–C (14)	1.393 (9)	C (24)–C (25)	1.369 (18)
C (2)–C (3)	1.403 (13)	C (3)–C (4)	1.333 (18)
C (17)–C (16)	1.369 (13)	C (13)–C (12)	1.392 (16)
**$(C_{13}H_{26}N)_2MnBr_4$**			
Atom–Atom	Length/Å	Atom–Atom	Length/Å
Br (01)–Mn (1)	2.4976 (9)	C (8)–C (13)	1.516 (11)
Br (02)–Mn (1)	2.5226 (9)	C (8)–C (9)	1.483 (10)
Br (03)–Mn (1)	2.5099 (9)	C (13)–C (12)	1.523 (13)
Br (04)–Mn (1)	2.4704 (9)	C (22)–C (23)	1.522 (11)
N (1)–C (14)	1.510 (6)	C (15)–C (16)	1.528 (10)
N (1)–C (21)	1.531 (6)	C (19)–C (18)	1.560 (11)
N (1)–C (20)	1.508 (6)	C (1)–C (2)	1.559 (15)
C (14)–C (15)	1.513 (8)	C (25)–C (24)	1.483 (12)
C (14)–C (19)	1.498 (8)	C (24)–C (23)	1.515 (12)
C (21)–C (26)	1.511 (7)	C (2)–C (3)	1.476 (17)
C (21)–C (22)	1.489 (8)	C (16)–C (17)	1.477 (14)
C (26)–C (25)	1.519 (8)	C (10)–C (9)	1.502 (11)
N (2)–C (6)	1.578 (10)	C (10)–C (11)	1.494 (15)
N (2)–C (8)	1.460 (9)	C (5)–C (4)	1.463 (15)
N (2)–C (7)	1.481 (9)	C (18)–C (17)	1.467 (15)
C (6)–C (1)	1.503 (9)	C (11)–C (12)	1.469 (15)
C (6)–C (5)	1.487 (10)	C (3)–C (4)	1.536 (14)

Table S2. The bond angles (°) of $(C_{13}H_{14}N)_2MnBr_4$ and $(C_{13}H_{26}N)_2MnBr_4$.

**$(C_{13}H_{14}N)_2MnBr_4$**			
Atom–Atom–Atom	Angle/°	Atom–Atom–Atom	Angle/°
Br (3)–Mn (1)–Br (1)	108.56 (4)	C (10)–C (9)–C (8)	118.5 (9)
Br (3)–Mn (1)–Br (4)	110.62 (4)	C (15)–C (14)–C (19)	117.9 (7)
Br (4)–Mn (1)–Br (1)	110.25 (5)	C (1)–C (6)–C (5)	118.5 (11)
Br (2)–Mn (1)–Br (3)	117.83 (6)	C (24)–C (23)–C (22)	120.2 (9)
Br (2)–Mn (1)–Br (1)	106.14 (5)	C (12)–C (11)–C (10)	119.2 (9)
Br (2)–Mn (1)–Br (4)	103.19 (5)	C (4)–C (5)–C (6)	119.5 (9)
C (19)–N (1)–C (20)	113.1 (6)	C (9)–C (8)–N (2)	116.3 (7)

C (19)–N (1)–C (21)	110.9 (5)	C (9)–C (8)–C (13)	122.4 (8)
C (21)–N (1)–C (20)	114.2 (6)	C (13)–C (8)–N (2)	121.3 (9)
C (7)–N (2)–C (1)	111.9 (8)	C (21)–C (22)–C (23)	118.0 (9)
C (8)–N (2)–C (1)	111.4 (5)	C (9)–C (10)–C (11)	121.4 (11)
C (8)–N (2)–C (7)	114.8 (7)	C (22)–C (21)–N (1)	121.2 (7)
C (19)–C (18)–C (17)	119.5 (7)	C (26)–C (21)–N (1)	116.4 (6)
C (2)–C (1)–N (2)	117.1 (7)	C (26)–C (21)–C (22)	122.4 (8)
C (2)–C (1)–C (6)	122.7 (8)	C (21)–C (26)–C (25)	119.0 (9)
C (6)–C (1)–N (2)	120.2 (8)	C (23)–C (24)–C (25)	121.1 (10)
C (18)–C (19)–N (1)	118.5 (6)	C (4)–C (3)–C (2)	120.9 (11)
C (18)–C (19)–C (14)	121.4 (7)	C (14)–C (15)–C (16)	121.6 (8)
C (14)–C (19)–N (1)	120.1 (6)	C (3)–C (4)–C (5)	120.3 (10)
C (1)–C (2)–C (3)	118.1 (9)	C (24)–C (25)–C (26)	119.2 (11)
C (16)–C (17)–C (18)	119.9 (8)	C (8)–C (13)–C (12)	117.7 (12)
C (17)–C (16)–C (15)	119.7 (8)	C (11)–C (12)–C (13)	120.9 (10)

$(C_{13}H_{26}N)_2MnBr_4$

Atom–Atom–Atom	Angle/°	Atom–Atom–Atom	Angle/°
Br (01)–Mn (1)–Br (02)	108.61 (3)	N (2)–C (8)–C (13)	110.4 (6)
Br (01)–Mn (1)–Br (03)	107.07 (3)	N (2)–C (8)–C (9)	115.6 (6)
Br (03)–Mn (1)–Br (02)	108.34 (3)	C (9)–C (8)–C (13)	110.3 (6)
Br (04)–Mn (1)–Br (01)	114.21 (4)	C (8)–C (13)–C (12)	115.6 (8)
Br (04)–Mn (1)–Br (02)	109.38 (4)	C (21)–C (22)–C (23)	110.0 (6)
Br (04)–Mn (1)–Br (03)	109.07 (4)	C (14)–C (15)–C (16)	110.4 (6)
C (14)–N (1)–C (21)	118.7 (4)	C (14)–C (19)–C (18)	109.3 (6)
C (20)–N (1)–C (14)	113.5 (4)	C (6)–C (1)–C (2)	109.0 (7)
C (20)–N (1)–C (21)	109.3 (4)	C (24)–C (25)–C (26)	114.3 (6)
N (1)–C (14)–C (15)	108.5 (4)	C (25)–C (24)–C (23)	113.3 (6)
C (19)–C (14)–N (1)	114.6 (4)	C (24)–C (23)–C (22)	110.3 (7)
C (19)–C (14)–C (15)	112.4 (5)	C (3)–C (2)–C (1)	113.3 (7)
C (26)–C (21)–N (1)	110.7 (4)	C (17)–C (16)–C (15)	113.3 (8)
C (22)–C (21)–N (1)	117.0 (4)	C (11)–C (10)–C (9)	114.4 (8)
C (22)–C (21)–C (26)	112.9 (5)	C (4)–C (5)–C (6)	112.1 (9)
C (21)–C (26)–C (25)	109.4 (5)	C (17)–C (18)–C (19)	111.6 (7)
C (8)–N (2)–C (6)	116.2 (5)	C (8)–C (9)–C (10)	116.1 (7)
C (8)–N (2)–C (7)	116.2 (7)	C (12)–C (11)–C (10)	114.6 (10)
C (7)–N (2)–C (6)	110.7 (5)	C (18)–C (17)–C (16)	111.6 (6)
C (1)–C (6)–N (2)	112.6 (6)	C (11)–C (12)–C (13)	112.6 (8)
C (5)–C (6)–N (2)	111.2 (6)	C (2)–C (3)–C (4)	111.0 (7)
C (5)–C (6)–C (1)	112.0 (6)	C (5)–C (4)–C (3)	111.5 (10)

Table S3. Main parameters of processing and refinement of $(C_{13}H_{14}N)_2MnBr_4$ and $(C_{13}H_{26}N)_2MnBr_4$.

**Compound**	**$(C_{13}H_{14}N)_2MnBr_4$**	**$(C_{13}H_{26}N)_2MnBr_4$**
Sp.Gr.	*P*1	$P2_1/c$
a, Å	8.51480(28)	11.62635(35)
b, Å	8.63446(29)	30.76613(89)
c, Å	10.66282(41)	9.53011(32)
α, °	87.0893(22)	90
β, °	67.2425(19)	104.9861(22)
γ, °	87.1786(24)	90
V, Å^3	721.603(45)	3292.96(18)
Z	4	4

2θ-interval, º	7-100	0-0
R_{wp}, %	3.33	3.35
R_p, %	2.58	2.52
χ^2	1.81	1.94
R_B, %	1.94	2.43
